# Occurrence and Exposure Assessment of Lipophilic Shellfish Toxins in the Zhejiang Province, China

**DOI:** 10.3390/md22060239

**Published:** 2024-05-24

**Authors:** Qin Weng, Ronghua Zhang, Pinggu Wu, Jiang Chen, Xiaodong Pan, Chenyang Zheng, Dong Zhao, Jikai Wang, Hexiang Zhang, Xiaojuan Qi, Junde Han, Zijie Lu, Biao Zhou

**Affiliations:** 1 School of Public Health, Hangzhou Medical College, Hangzhou 310013, China; 881012022119@hmc.edu.cn (Q.W.); 130232023409@hmc.edu.cn (Z.L.); 2 Zhejiang Provincial Center for Disease Control and Prevention, Hangzhou 310051, China; rhzhang@cdc.zj.cn (R.Z.); pgwu@cdc.zj.cn (P.W.); jchen@cdc.zj.cn (J.C.); xdpan@cdc.zj.cn (X.P.); cyzheng@cdc.zj.cn (C.Z.); dzhao@cdc.zj.cn (D.Z.); jkwang@cdc.zj.cn (J.W.); hxzhang@cdc.zj.cn (H.Z.); xjqi@cdc.zj.cn (X.Q.); 3 Department of Epidemiology and Health Statistics, School of Public Health, Faculty of Medicine, Hangzhou Normal University, Hangzhou 311121, China; 2021112012151@stu.hznu.edu.cn

**Keywords:** lipophilic shellfish toxins, diarrheic shellfish toxins, dietary exposure, risk assessment, seafood safety

## Abstract

Although lipophilic shellfish toxins (LSTs) pose a significant threat to the health of seafood consumers, their systematic investigation and risk assessment remain scarce. The goals of this study were as follows: (1) analyze LST levels in commercially available shellfish in Zhejiang province, China, and determine factors influencing LST distribution; (2) assess the acute dietary risk of exposure to LSTs for local consumers during the red tide period; (3) explore potential health risks of LSTs in humans; and (4) study the acute risks of simultaneous dietary exposure to LSTs and paralytic shellfish toxins (PSTs). A total of 546 shellfish samples were collected. LSTs were detected in 89 samples (16.3%) at concentrations below the regulatory limits. Mussels were the main shellfish species contaminated with LSTs. Spatial variations were observed in the yessotoxin group. Acute exposure to LSTs based on multiple scenarios was low. The minimum tolerable exposure durations for LSTs calculated using the mean and the 95th percentile of consumption data were 19.7 and 4.9 years, respectively. Our findings showed that Zhejiang province residents are at a low risk of combined exposure to LSTs and PSTs; however, the risk may be higher for children under 6 years of age in the extreme scenario.

## 1. Introduction

In recent years, harmful algal blooms have frequently occurred worldwide [1,2]. Toxins produced by harmful algal species accumulate in shellfish through the food chain and cause seafood poisoning events, raising concerns about the quality of shellfish products and their danger to public health. Marine toxins can be categorized as lipophilic or hydrophilic, based on their solubility [3]. Hydrophilic toxins are distributed globally [4,5,6,7,8], especially saxitoxin (STX) and domoic acid toxin groups, which cause paralytic and amnesic shellfish poisoning, respectively [9,10,11,12]. However, more than 90% of marine toxins are lipophilic [13], with examples including okadaic acid (OA) and its analogs dinophysistoxins (DTXs), pectenotoxins (PTXs), azaspiracids, yessotoxins (YTXs), spirolids, gymnodimines, and pinnatoxins, collectively known as lipophilic shellfish toxins (LSTs) [14,15]. Contamination of shellfish with LSTs has been reported in the coastal areas of many countries, including China [16,17], Chile [18], Australia [19], the United States of America [20], the United Kingdom [21], and African countries [22]. Given that aquaculture production is growing globally, the increasing incidence of shellfish contamination by LSTs [23] is of great concern owing to the risk it poses to the health of seafood consumers.

LSTs and phytoplankton species producing them have been frequently reported in recent years [23]. Among these, the OA group toxins are considered the predominant toxins in China, as they have been isolated from various species of bivalve shellfish [24,25]. Other LSTs, such as YTXs and PTX2, have also been detected frequently [24,26]. PTX2, OA, and its derivatives (DTX1 and DTX2) are heat-stable polyether compounds widely reported in *Dinophysis* spp. [26,27,28,29,30,31]. YTXs are a group of structurally related polyether toxins produced by the dinoflagellates *Protoceratium reticulatum*, *Lingulodinium polyedrum*, and *Gonyaulax spinifera* [32]. Currently, most reports on these toxins mainly focus on their acute rather than chronic toxicity [29,30,33]. For example, acute shellfish poisoning may occur with symptoms of diarrhea, nausea, vomiting, and abdominal pain, within 30 min to several hours after human consumption of shellfish containing high levels of OA and DTXs [29]. PTX2 has low toxicity in mice after oral ingestion but is acutely toxic after an intraperitoneal injection [34].

Some highly lipophilic substances, such as persistent organic pollutants and pesticides, accumulate in human adipose tissue over time [35]. It is plausible that LSTs also accumulate in the human body and present a health hazard. For example, OA analogs inhibit protein phosphatases and promote tumor formation in mouse skin, rat glandular stomach, and rat liver [36]. OAs also show neurotoxicity in zebrafish [37] and rats [38]. YTXs compromise cardiovascular function in rats and change heart ultrastructure, which suggests that repeated exposure to YTXs may be also hazardous to humans [39,40]. With global climate change and marine eutrophication, the levels of toxic algae are likely to increase, which would, in turn, elevate concentrations of marine algal toxins [41,42,43]. Therefore, not only does the risk of acute poisoning by shellfish toxins increase but so does the risk of chronic poisoning, thus requiring greater attention.

Contamination of shellfish by LSTs has become a major concern for public health authorities and the shellfish cultivation industry globally because of the impact of LSTs on seafood safety and human health [44,45]. Chinese national standards set a combined regulatory limit for OA and DTX levels in shellfish at 160 µg OA eq./kg (µg OA equivalents/kg) [46], which is consistent with that of the European Union (EU) [47]. However, there are currently no relevant regulations in China for other LSTs, including YTXs and PTXs. According to the EU regulation (EC) no. 853/2004, the regulatory limit for YTXs is 3.75 mg YTX eq./kg [47]. PTXs used to be considered together with OA and DTXs, but these were recently exempted from the regulations [47,48,49]. The European Food Safety Authority (EFSA) established an acute reference dose (ARfD) for LSTs to prevent accidents caused by the ingestion of contaminated shellfish. In particular, ARfDs of 0.3 µg OA eq./kg body weight (b.w.) for OA group toxins, 0.8 µg PTX2 eq./kg b.w. for PTX group toxins, and 25 µg YTX eq./kg b.w. for YTX group toxins are recommended [50]. Notably, despite the fact that LSTs can persist at low concentrations in shellfish over long periods of time [24,26,51], and chronic symptoms can occur upon long-term consumption of shellfish [52,53], reference doses suitable for gauging chronic dietary exposure of humans to LSTs have not been established [26]. Thus, a non-carcinogenic health risk assessment model and ARfDs were used to assess the chronic risk of exposure to LSTs in the present study.

Shellfish cultivation is an important industry in China, especially in Zhejiang in the southeast of China, where local shellfish are potentially at risk of contamination with LSTs. The cumulative number and area of red tides in Zhejiang province increased between 2012 and 2017 [54]. On 27 May 2011, a foodborne incident occurred in Zhejiang province during which more than 220 cases of suspected or probable acute diarrheic shellfish poisoning were detected, with OA group toxins in shellfish exceeding the regulatory limits [16]. However, comprehensive investigations of dietary exposure to LSTs in Zhejiang remain limited. Only a few studies related to LSTs have been conducted in Zhejiang province [55], which however did not properly address LST contamination and exposure risk for Zhejiang residents. The species, time, and sites of shellfish sampling [3,24,26,56,57], algal blooms [58], and cooking progress [25] add to the complexity of toxin detection in shellfish and dietary assessment. The concentrations and distributions of LSTs in shellfish in Zhejiang and the dietary exposure of local residents to LSTs remain to be determined.

Therefore, the objectives of this study were to analyze six types of LSTs (OA, DTX1, DTX2, YTX, homoYTX, and PTX2) in commercially available shellfish in Zhejiang province, establish factors influencing LST distribution, assess the acute risk of dietary exposure to LSTs for local consumers during the red tide frequency period, explore the potential LST-associated health risk to humans, and estimate the probability of simultaneous exposure to both LSTs and paralytic shellfish toxins (PSTs) in conjunction with a recently published article on the assessment of dietary exposure to PSTs in Zhejiang province [59]. The data generated in this study also provide a scientific basis for future assessments of the chronic risk of exposure to LSTs.

## 2. Results

### 2.1. Occurrence of LSTs

We collected 546 shellfish samples in Zhejiang province, including those of *Atrina pectinata* (n = 2), *Scapharca subcrenata* (n = 24), *Arcidae* (except *Scapharca subcrenata*) (n = 53), oysters (n = 77), scallops (n = 36), and mussels (n = 354). Five distinct types of LSTs (OA, DTX-1, PTX2, homoYTX, and YTX) were detected in eighty-nine samples (16.3%). Only two *Atrina pectinata* were collected in 2018 because of the low production of this species in Zhejiang province [60]. HomoYTX was detected most frequently and at the highest concentrations (Table 1). OA and YTX group toxins were simultaneously detected in three samples.

These toxins can be categorized into three groups based on their indicator compounds. Non-detected (ND) results were substituted with 0 and the limit of detection (LOD) to derive the lower (LB) and upper (UB) bounds of concentrations. Detailed descriptive statistics of the detection rates and concentrations of the three LST groups from different subgroups (sampling species, sampling time, and sampling sites) are summarized in Tables S1–S3. In this study, LSTs were detected in mussels (85/354), scallops (3/36), and oysters (1/77). No toxins were detected in the two *Atrina pectinate*, twenty-four *Scapharca subcrenata*, and fifty-three *Arcidae* (except for *Scapharca subcrenata*) samples.

OA group toxins were detected in mussels from two sampling sites. One toxin-positive sample was detected in August 2018, and seven were detected in June 2019. Mussels collected from Ningbo in August 2018 contained the highest concentrations of OA group toxins, ranging from 16.5 (LB) to 32.5 (UB) µg OA eq./kg.

PTX2 was detected in two species, oysters and mussels, collected from Wenzhou in June 2018. The maximum concentration of PTX2 was 13 µg PTX2 eq./kg.

The YTX group toxins were mainly detected in mussels (79/354), followed by scallops (3/36), and with no significant differences observed in the detection frequency between these (*p* > 0.05). The toxin detection frequency in samples collected from the five sampling sites was significantly different (*p* < 0.05). The concentrations of YTX group toxins were highest in June. Mussels from Wenzhou in June 2019 contained the highest concentrations of YTXs, ranging from 373 (LB) to 393 (UB) µg YTX eq./kg.

### 2.2. Co-Occurrence of LSTs and PSTs in Shellfish in Zhejiang Province

Out of 546 bivalve shellfish samples collected in Zhejiang province in 2018–2019 for the present study, 119 samples contained at least one type of marine toxin, 81 samples contained LSTs only and 30 samples contained PSTs only. Furthermore, eight samples were co-contaminated with LSTs and PSTs, of which seven samples were from the mussels collected in June 2019.

### 2.3. Dietary Exposure Assessment

#### 2.3.1. Acute Exposure to LSTs

The acute dietary exposure values and %ARfD for each LST group are presented in Table 2. In scenario 1, shellfish consumption and body weight data obtained from the Food Consumption Survey of Zhejiang province were used for the risk assessment. The dietary exposure values for Zhejiang province consumers were 0.02 (LB)–0.05 (UB) µg OA eq./kg b.w., 0.02 µg PTX2 eq./kg b.w., and 0.55 (LB)–0.58 (UB) µg YTX eq./kg b.w. for OA, PTX, and YTX group toxins, respectively. Meanwhile, the %ARfD for each LST group was far below 100% in all age groups, with the ARfD% in each group decreasing in the following order: OA > PTX > YTX. Children ≤ 6 years of age were at a higher risk of dietary exposure to toxins than individuals in other age groups. In scenario 2, dietary exposure was assessed using shellfish consumption data (400 g) and the body weight of adults (60 kg) as proposed by the Panel on Contaminants in the Food chain (CONTAM Panel) [29]. The calculated exposure value was roughly 4.5 times higher than that calculated by our method but still within the safe range. In scenario 3, the data were from Korea [6], which is also in Eastern Asia, and the dietary exposure results were also similar to ours.

**Figure 1:**
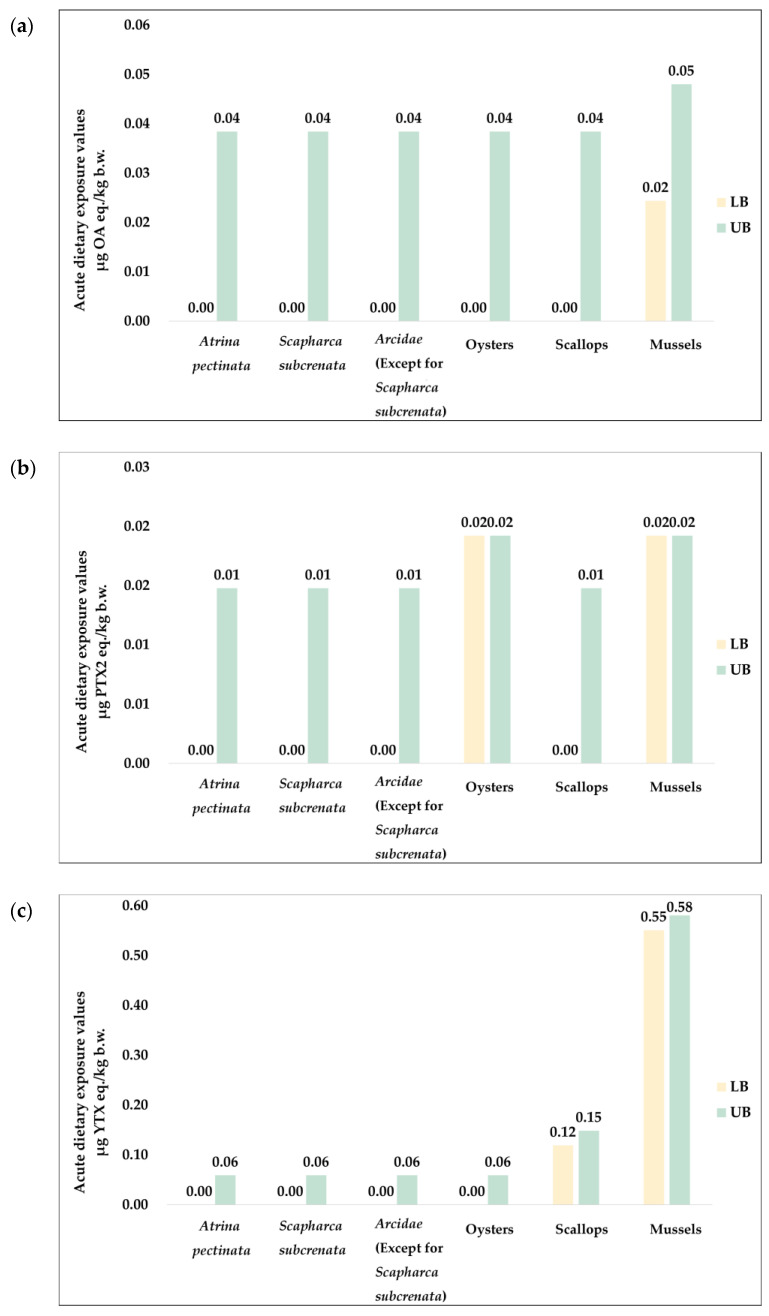
Acute dietary exposure to three groups of LSTs from each shellfish species. (**a**) OA group toxins; (**b**) PTX group toxins; (**c**) YTX group toxins. LB: ND = 0; UB: ND = LOD.

Regarding the contribution of different shellfish species to the acute dietary exposure values of toxins in each group (Figure 1), mussels were the main source of OA (20–100%) and YTX (60.1–82.2%) group toxins. In addition, scallops contributed 15.4–17.8% of YTX group toxins. For PTX group toxins, mussels and oysters contributed equally (50% when ND = 0 and 19.7% when ND = LOD).

#### 2.3.2. Tolerable Duration of Non-Carcinogenic LST Exposure

The LB and UB of concentrations in shellfish samples were used to assess the tolerable duration of non-carcinogenic LST exposure via shellfish consumption by Zhejiang province consumers based on different scenarios (Table 3). When the mean consumption data were used (scenario 1), the tolerable durations of exposure to LSTs were 19.7–2839.3 years, 136.7–28,742.4 years, and 848.3–3186.2 years for the OA, PTX2, and YTX group toxins, respectively. When the 95th percentile shellfish consumption data were used to simulate an extreme situation, tolerable durations of exposure to LSTs comprised just 24.7% of those predicted by scenario 1. The lowest tolerable exposure duration (4.9 years) was predicted for OA group toxins in scenario 2. When LB values were used, the tolerable durations of exposure were as follows: OA < PTX2 < YTX group. The use of UB values modified this order: OA < YTX < PTX group.

#### 2.3.3. Acute Combined Dietary Exposure to LSTs and PSTs

Given that LSTs usually co-occur with PSTs in shellfish, the hazard index (HI) values of the acute dietary exposure to LSTs alone and LSTs plus PSTs were calculated (Table 4). From the perspective of exposure to LSTs alone, the HI obtained by summing the %ARfD values of the OA, PTX, and YTX group toxins was less than 100% in scenarios 1 and 2, indicating that the population is at a low risk of simultaneous exposure to all three groups of LSTs. The addition of PSTs increased the HI value above 100% (in the UB scenario) for most people in Zhejiang province, especially for children under 6 years of age, suggesting that they might be at risk (scenario 1). We also used the 95th percentile concentrations for the calculations to avoid the effects of extreme values. The risk of combined exposure to LSTs and PSTs in the general population of consumers ranged from 2.5% to 55.2% (scenario 2), with only children under 6 years of age being at a risk of exposure, which is a more optimistic prediction than that in scenario 1.

## 3. Discussion

The presence of LSTs in shellfish adversely affects the prospects of mariculture and the seafood industry. The detection rate of LSTs in shellfish from Zhejiang province (16.3%) was modest compared with that in Shenzhen (approximately 33.3%) [26], Guangxi (82.9%) [57], and Fujian (11.3%) [61], which are also located in southeastern China. Furthermore, a high rate of LST detection (more than 95%), albeit at concentrations not exceeding the regulatory limits, was also reported in the Bohai Sea region of China, which could be explained by the presence of toxic algae in seawater [56]. Shellfish collected from fisheries in Scotland closed due to the detection of algal toxins during routine monitoring had an LST detection rate of 63% [62]. In terms of the profile of LSTs, previous studies conducted in the Fujian province (detection rate: 8.4%, maximum concentration: 429 µg/kg) [24] and Shenzhen (22.3%, 50 µg/kg) also found that the dominant toxin was homoYTX, which is consistent with the results of our present study (15.0%, 373 µg/kg). However, other studies have reported different results. In Guangxi, gymnodimines, OA, and DTX2 were detected at higher frequencies (83.02%, 51.16%, and 40.91%, respectively), while homoYTX was detected only in 8.60% of the samples (maximum concentration: 159.66 µg/kg) [57]. Around the Bohai Sea region of China, YTX was the most dominant LST in shellfish samples from Laishan and Laizhou, while PTX2 and 13–desmethyl spirolide C were the predominant LSTs detected in Qinhuangdao, Hangu, and Huludao, which may have been caused by the presence of potentially toxic microalgae [56]. In addition, Pearson correlation analysis showed that environmental factors such as water depth, dissolved oxygen, particulate silicon, temperature, and pH all affect the composition and distribution of LSTs in seawater [3] and, consequently, in shellfish.

In the present study, the maximum concentration range of the OA group toxins was 16.5–32.5 µg OA eq./kg, which was lower than the regulatory limit of 160 µg OA eq./kg [47], and similar to the concentration reported in samples from Shenzhen (maximum concentration: 25–34.6 µg OA eq./kg) [26], the Middle Adriatic Sea (44.7 µg OA eq./kg) [63], and the Argentine Sea (12.9 µg OA eq./kg) [64]. The highest concentration of PTX2, which was recently deregulated [47,48,49], was 13 µg PTX2 eq./kg, similar to that detected in samples from Shenzhen (6 µg PTX2 eq./kg) [26] and Hebei (10–30 µg PTX2 eq./kg) [65]. The maximum concentration of YTX group toxins was 0.373–0.393 mg YTX eq./kg, lower than the regulatory limit of 3.75 mg YTX eq./kg [47]. The range of YTX group toxin concentrations detected was higher than that of samples from Shenzhen (50–52 µg YTX eq./kg) [26] and Hebei (40–80 µg YTX eq./kg) [65], but lower than that reported in samples from Great Britain (1800 µg YTX eq./kg) [66]. During these routine monitoring periods, toxin levels were not high, remaining below the regulatory limits. However, during shellfish poisoning outbreaks, OA group toxins can be as high as 338 µg OA eq./kg [67], suggesting that regular monitoring is crucial because although these toxins may be present at low levels, they can persist in the environment for long periods.

In the present study, LSTs, particularly YTX group toxins, were predominantly detected in mussels. The reason for this higher detection rate may be explained by the greater capacity of mussels to accumulate toxins relative to that of other shellfish species [24,25,68]. Mussels are one of the best indicator species for diarrheic shellfish toxins [69] and PSTs [70] in some areas. Previous studies showed that oysters and clams generally contain less than half the amount of toxins detected in mussels [20], which contain at least 10 times more diarrheic shellfish toxins than oysters [71]. In addition, the occurrence of YTXs in mussels was characterized by a seasonal pattern, with the maximum concentrations of YTXs in Fujian occurring in July [24], consistent with our results. In this study, the high detection rate of YTX in bivalves may have been affected by toxic red tides, which occurred from June to August in 2018 and from April to June in 2019 in Zhejiang waters [72,73]. Further, spring tides are associated with the harvest of larger submerged bivalves, and intertidal mussels grow larger [44]. Therefore, reduction of shellfish consumption during red tides is strongly recommended.

Out of all the sampling sites in this study, Hangzhou had a relatively high LST detection rate and was dominated by the presence of YTX group toxins. However, to the best of our knowledge, most shellfish in Hangzhou are not produced locally. In addition, LSTs are widespread in marine waters and usually present at low concentrations for long periods [24,26,56,57]. These toxins are detected in seawater samples and in various algal species [56,74]. Many toxin-producing algae species are present in coastal waters, and the composition of the toxins produced by phytoplankton varies considerably among different locations, being affected by environmental factors [3]. Further studies are needed to assess the composition and distribution of algae containing LSTs in the East China Sea region.

Acute dietary exposure to the three groups of toxins was determined using consumption and body weight data obtained from the Food Consumption Survey in Zhejiang province (scenario 1), the CONTAM Panel (scenario 2), and the Korea National Health and Nutrition Examination Survey (KNHANES) 2010–2015. All exposure values were within the safe ranges. Various studies have also assessed the dietary exposure to LSTs. In scenario 1, the calculated values of acute exposure to OA group toxins for residents of Zhejiang province (0.02–0.05 µg OA eq./kg b.w.; this study), Guangxi province (0.26 µg OA eq./kg b.w.) [57], and Shenzhen (0.065–0.09 µg OA eq./kg b.w.) [26] were relatively low. Even in scenario 2, the exposure values to OA group toxins for residents of Zhejiang province (0.11–0.22 µg OA eq./kg b.w.) was much lower than that for the residents of Guangxi province (0.34 µg OA eq.mg/kg b.w.), which was slightly higher than the safe range [57]. The acute exposure levels to PTX group toxins were also low for residents of Zhejiang province (0.02 µg PTX2 eq./kg b.w.) in comparison with those for residents of Shenzhen (0.016–0.02 µg PTX2 eq./kg b.w.) [26] and New Zealand (0.53 µg PTX2 eq./kg b.w.) [75]. The acute exposure levels to YTX group toxins were modest for Zhejiang province residents (0.55–0.58 µg YTX eq./kg b.w.) and comparable to those for residents of Guangxi province (0.60 µg YTX eq./kg b.w.) [57] and Shenzhen (0.13–0.14 µg YTX eq./kg b.w.) [26]. In summary, the potential risk of dietary intake of these toxins by residents of Zhejiang province is acceptable compared to the results from other areas.

In this study, the amount of toxin ingested with food was considered equal to the amount of toxin that can be absorbed by the human body, which could possibly overestimate the degree of the chronic exposure. In addition, it was shown that the bioaccessibility of OA group toxins varies by species, ranging from 88% in mussels to 75% in clams [10,76,77]. When this value is introduced into the calculation (i.e., when the aforementioned number of years is divided by the bioaccessibility), the minimum tolerable exposure duration in scenario 1 (19.7 years) becomes 22.4–26.3 years, and that in scenario 2 (4.9 years) becomes 5.6–6.5 years.

When the two classes of toxins were considered separately, the risk of exposure to PSTs was only present in children ≤ 6 years of age under the extreme scenario [59]. However, considering the co-occurrence of these toxins, the HI was calculated by summing the %ARfD values of the LSTs (toxins in the OA, PTX, and YTX groups) and PSTs, which showed that Zhejiang province residents were at a low risk of combined exposure to LSTs and PSTs, which may be higher for children ≤ 6 years of age in an extreme scenario. Compared with the results of the study of Scotland seawater, where the toxin detection rate was 4.4% (16/366) [62], the rate of 1.5% (8/546) of shellfish samples being contaminated with both LSTs and PSTs in the present study is relatively low. Meanwhile, we believe that the results of scenario 2 (calculated using the 95th percentile concentration instead of the maximum concentration) are more realistic and relevant to the actual situation. In addition, LSTs act synergistically under certain conditions [78,79], highlighting the need to consider interactions between PSTs and LSTs. However, because such interactions are rarely examined, investigating them further in future studies is necessary.

This study had some limitations. First, shellfish samples were collected during the high red tide season, meaning that the concentration data were likely to be higher than usual. Second, shellfish contamination levels were from the 2018–2019 survey, whereas consumption data were from the 2015–2016 survey. Finally, ARfD was used as the reference value for potential risk assessment, which is generally greater than or equal to the acceptable daily intake, a value often used for long-term dietary exposure assessment [80,81], which may have led to potential overestimation of the potential risk in the present study.

## 4. Materials and Methods

### 4.1. Sample Preparation

Shellfish samples were collected in duplicate from May 2018 to September 2018 and from May 2019 to September 2019 from representative and typical seafood markets in Zhejiang province by trained investigators. The sample size was determined using the following formula [82]:

(1) $$N=\frac{Z^{2}\times \left[P\times \left(1-P\right)\right]}{d^{2}}$$

where N is sample size; Z = 1.96 (95% confidence level); P = 0.5 (expected percentage of samples containing toxins); and d = 10%, representing precision. In this study, 546 shellfish samples were collected, including *Atrina pectinata* (n = 2), *Scapharca subcrenata* (n = 24), *Arcidae* (except *Scapharca subcrenata*) (n = 53), oysters (n = 77), scallops (n = 36), and mussels (n = 354). Details of the sampling sites and sample preparation methods were described in another published article [59].

### 4.2. Chemical Reagents

Acetonitrile and methanol were purchased from Merck (Darmstadt, Germany). Ammonia (25–28%) was purchased from CNW Technologies GmbH (Düsseldorf, Germany). Water was distilled and purified using a Millipore purification system (Millipore Ltd., Bedford, MA, USA). Certified reference materials for LSTs, including OA, DTX1, DTX2, YTX, homoYTX, and PTX2, were purchased from the National Research Council Certified Reference Materials Program (Institute for Marine Biosciences, Halifax, NS, Canada).

### 4.3. Sample Analysis

Local laboratories in Zhejiang province detected multiple LSTs in shellfish samples. Six types of LSTs were identified as previously described, with some modifications [24,26]. Briefly, 2 g of the homogenized specimen was extracted with 9 mL of methanol. The supernatant was then transferred to a 20 mL volumetric flask. Extraction was repeated with methanol, and the supernatants were combined in a final 20 mL volume of methanol. The resulting solution was passed through a 0.22 μm filter for subsequent analysis.

When free OA, DTX1, and DTX2 were not detected, the total LST amount was not determined. If at least one toxin was detected, the total LST amount was determined after alkaline saponification. In particular, 1 mL of the above-mentioned extract was mixed with 125 µL of 2.5 mol/L NaOH, heated to 76 °C for 40 min, and then cooled to room temperature. Thereafter, 125 µL of 2.5 mol/L hydrochloric acid and 2.5 mL of water were added to the mixture. After removal of impurities, the resulting solution was filtered through a 0.22 µm filter membrane and the concentration of the test substance was measured.

A Waters Xevo TQ-XS triple quadrupole mass spectrometer detector (Waters Corporation, Milford, MA, USA) equipped with an electrospray ionization source was used for mass spectrometry analysis. Chromatographic separation was performed with an Acquity BEH-C18 column (1.7 µm, 2.1 × 100 mm) at a column temperature of 40 °C. Mobile phase A was 0.01% ammonia and mobile phase B was acetonitrile. A gradient elution program was set up with an injection volume of 5 µL and a flow rate of 0.35 mL/min, starting with 20% mobile phase B.

### 4.4. Method Validation and Quality Control

All the laboratory staff involved were trained in the use of standardized experimental methods. Parameters such as accuracy, precision, linearity, and LOD of the experiments were validated by local laboratories, and qualified data were included in the contaminant database. The LOD was calculated as 3× signal-to-noise ratios. In the present study, the LOD values were 20 µg/kg for homoYTX and YTX and 10 µg/kg for OA, DTX-1, DTX-2, and PTX2.

### 4.5. Contamination Data Processing

OA, PTX2, and YTX were used as index compounds. The concentration of each LST was multiplied by the corresponding toxicity equivalence factor (TEF) and converted into equivalent concentrations of the index compounds. Cumulative equivalent concentration sums for each toxin group were calculated for each shellfish sample [6,26], and the units were µg OA eq./kg, µg PTX2 eq./kg, and µg YTX eq./kg, respectively. The relevant TEFs and regulatory limits are presented in Table 5.

Left-censored data were treated using the substitution method [83,84], that is, concentrations of all ND were set to zero in the LB scenario, whereas in the UB scenario, ND was set to the LOD of each toxin [26].

### 4.6. Food Consumption Data

Shellfish consumption data were obtained from the Food Consumption Survey of Zhejiang province between 2015 and 2016. This survey was conducted in 10 cities in Zhejiang province using 3 non-consecutive, 24 h dietary recall face-to-face interviews and food frequency questionnaires. All participants signed an informed consent form and their personal information was kept confidential.

Approximately 19,968 residents aged 3 years and older completed 3 non-consecutive, 24 h dietary recall face-to-face interviews. Next, 1075 individuals that consumed various bivalve shellfish without missing information (such as age and weight) were selected. Food consumption data obtained were analyzed as previously described [84] by considering valid consumer days of consumers only as independent observations in the database without averaging when calculating the consumption percentile to obtain a large portion size [9,50]. This approach has been described by us previously [59].

Furthermore, food frequency questionnaires were also completed by the residents of Zhejiang province to determine their shellfish consumption frequency and rates during this period. A total of 8265 residents aged 3 and older without missing information (such as age and weight) provided such information. Using food frequency questionnaires, we also obtained the median annual frequency of shellfish consumption by these consumers, which was 12 days per year.

These participants were categorized into five groups: young children (≤6 years), older children (7–13 years), adolescents (14–17 years), adults (≥18 years), and older adults (≥60 years) [59], as detailed in Section 2.

### 4.7. Dietary Exposure Assessment

#### 4.7.1. Acute Dietary Exposure

Point-estimate modeling was used to assess acute dietary exposure to LSTs among Zhejiang residents [26,84], and the general equation for calculating dietary exposure values was as follows:

(2) $$\begin{array}{l} \mathrm{Dietary}\;\mathrm{exposure}\;({µg\;\mathrm{kg}}^{-1}\;b.w.{\mathrm{day}}^{-1})\\ \;\;\;\;\;\;\;=\frac{\mathrm{Concentration}\;\mathrm{of}\;\mathrm{LSTs}\;\mathrm{in}\;\mathrm{shellfish}\;\mathrm{meat}\;\left(\text{µ}g\cdot {\mathrm{kg}}^{-1}\right)\times \mathrm{shellfish}\;\mathrm{meat}\;\mathrm{consumption}\;\left(g\cdot d^{-1}\right)}{\mathrm{Body}\;\mathrm{weight}\;\left(\mathrm{kg}\right)}\times 10^{-3} \end{array}$$

LST concentrations were derived from the analyzed shellfish sample data. Food consumption and body weight were used for calculating three high consumption scenarios to assess acute dietary exposures. In particular, the 95th percentile of daily shellfish consumption and mean body weight of Zhejiang province consumers obtained through three non-consecutive, 24 h dietary recall face-to-face interviews were used for scenario 1 [59]. The 95th percentile of daily shellfish consumption (400 g) and body weight (60 kg) of the adult population, as recommended by the CONTAM Expert Panel, were used for scenario 2 [29]. Finally, the 95th percentile of daily shellfish consumption (88 g) and body weight (58.5 kg) of the consumers from the KNHANES 2010–2015 were used for scenario 3 [6].

The acute health risk of LSTs was assessed by calculating %ARfD through dividing the estimated exposure to LSTs in shellfish by the corresponding ARfD. When %ARfD ≤ 100%, the risk of acute dietary intake was considered acceptable. Conversely, when %ARfD > 100%, the risk was considered unacceptable [85].

#### 4.7.2. Tolerable Duration of Exposure to Non-Carcinogenic LSTs

When assessing the non-carcinogenic risk of shellfish in the general population of Zhejiang consumers, the mean (scenario 1) and the 95th percentile (scenario 2) shellfish consumption data and the mean body weight (57.9 kg) obtained from the food frequency questionnaires were used in conjunction with the mean LST levels, as per the following equation [86]:

(3) $$\mathrm{THQ}=\frac{C\times \mathrm{FIR}\times \mathrm{EF}\times \mathrm{ED}}{\mathrm{BW}\times \mathrm{AT}\times \mathrm{RfD}}$$

where THQ is the target hazard quotient (dimensionless), C is the concentration of LSTs in shellfish meat (µg/kg), FIR represents the daily shellfish consumption rate in Zhejiang province (g person^−1^d^−1^), EF is exposure frequency (12 days/year), ED is exposure duration, BW is the average body weight (kg), AT is the average time for non-carcinogens (365 days/year × ED [87]), and RfD represents an estimate of the amount of daily exposure that an individual can continuously have over a lifetime without a significant risk of harmful effects. However, only EFSA-recommended ARfDs (µg toxin equivalents/kg b.w.) for each group of toxins were available. If the THQ is <1, significant adverse effects are unlikely to occur in the exposed population. If the THQ is ≥1, there is a potential health risk from significant adverse effects [86]. Assuming THQ = 1, the ARfD for each toxin group was divided by the dietary exposure value for the shellfish toxin group to which the population was exposed at an exposure frequency of 12 days/year for 1 year to derive the tolerable exposure duration.

#### 4.7.3. Hazard Index

HI values represent the combined risk of multiple shellfish toxins [88]. However, the interaction mechanisms and coefficients of correlation between these toxins remain unknown. In our study, the HI was calculated by summing the %ARfD values of each group of shellfish toxins derived from deterministic estimates. The acute dietary exposure (95th percentile consumption and maximum concentration) of Zhejiang province consumers to shellfish toxins was integrated and set as scenario 1. At the same time, the 95th percentile concentration of each toxin group was also introduced to avoid the effect of extreme values (scenario 2). Generally, an HI of less than 1.0 indicates that people are unlikely to be exposed to toxic levels that could have health consequences. Conversely, if the HI exceeds 1.0, there is a potential for adverse effects.

### 4.8. Statistical Analysis

Statistical analyses were performed using IBM SPSS software (version 25.0; IBM Corp., Armonk, NY, USA). Quantitative data are presented as means, medians, and 95th percentiles. Enumeration data are presented as rates. Chi-squared and Fisher’s exact tests were used to determine whether the detection rates of LSTs in shellfish depended on the sampling species, site, or time. The Bonferroni correction for multiple testing was applied for partially correlated measurements. Effects were considered statistically significant if *p* < 0.05.

## 5. Conclusions

This study describes the occurrence and distribution of LSTs in commercial shellfish products collected from Zhejiang in 2018–2019, estimates the acute and potential dietary exposure of local residents, and explores the acute dietary risks of exposure to LSTs alone or in combination with PSTs in Zhejiang province consumers. Five individual LSTs (OA, DTX1, PTX2, YTX, and homoYTX) were identified in shellfish samples, and homoYTX was the most frequently detected toxin. Mussels were the main shellfish species contaminated with LSTs. Spatial variations in LST distribution were observed in the YTX group. The results of the acute dietary exposure assessment indicated that the risk of exposure to LSTs through the consumption of bivalves is low. The risk of long-term exposure to LSTs also needs to be considered, and bivalve consumption needs to be reduced during periods of high red tides. The risk of concurrent exposure to LSTs and PSTs is low in the general population of Zhejiang province; however, children ≤ 6 years of age are potentially at risk. Notably, despite the fact that LSTs are observed at low levels in shellfish, they persist for long periods and no suitable reference dose for chronic exposure to LSTs has been established. Studies on interactions of LSTs with other toxins are scarce. Thus, the data from the current study may provide a reference for the formulation of future regulatory policies.

## Figures and Tables

**Table 1:** Occurrence and concentration ranges of six individual LSTs in the analyzed samples (n = 546).

Individual LSTs	N ^1^	Concentration Range (<LOD–Max, µg/kg)
OA	4	<10–16
DTX1	4	<10–16.5
DTX2	0	ND ^2^
PTX2	2	<10–13
YTX	3	<20–80.6
HomoYTX	79	<20–373

^1^ N, the number of samples above the limit of detection (LOD); ^2^ ND, non-detected.

**Table 2:** Acute dietary exposure and %ARfD of each type of LSTs in different age groups under various scenarios.

Scenario	Age (Years)	N	Body Weight	P95 Shellfish Consumption (g/d)	Dietary Exposure LB ^4^–UB ^5^ (µg/kg b.w.)			%ARfD ^6^ LB–UB (%)		
					OA	PTX2	YTX	OA	PTX2	YTX
Scenario 1 ^1^	All	1075	57.9	85.5	0.02–0.05	0.02	0.55–0.58	8.1–16.0	2.4	2.2–2.3
	≤6	50	19.2	64.0	0.05–0.11	0.04	1.24–1.31	18.3–36.0	5.4	5.0–5.2
	7–13	83	35.4	58.5	0.03–0.05	0.02	0.62–0.65	9.1–17.9	2.7	2.5–2.6
	14–17	22	57.7	78.9	0.02–0.04	0.02	0.51–0.54	7.5–14.8	2.2	2.0–2.1
	18–59	817	62.0	92.0	0.02–0.05	0.02	0.55–0.58	8.2–16.1	2.4	2.2–2.3
	≥60	103	62.2	71.0	0.02–0.04	0.01	0.43–0.45	6.3–12.4	1.9	1.7–1.8
Scenario 2 ^2^	Adult	-	60.0	400.0	0.11–0.22	0.09	2.49–2.62	36.7–72.2	10.8	9.9–10.5
Scenario 3 ^3^	All	-	58.5	88.0	0.02–0.05	0.02	0.56–0.59	8.3–16.3	2.4	2.2–2.4

^1^ Scenario 1, maximum contamination level of the samples combined with the 95th percentile (P95) daily shellfish consumption and mean body weight in the Zhejiang population. ^2^ Scenario 2, maximum contamination level of the samples combined with the 95th percentile (P95) daily shellfish consumption and body weight of adults proposed by the Panel on Contaminants in the Food chain (CONTAM Panel) [29]. ^3^ Scenario 3, maximum contamination level of the samples combined with the 95th percentile (P95) daily shellfish consumption and body weight of the consumers only from the Korea National Health and Nutrition Examination Survey (KNHANES) 2010–2015 [6]. ^4^ LB, lower bound, ND = 0; ^5^ UB, upper bound, ND = LOD. ^6^ %ARfD, the percentage of exposure to the ARfD (acute reference dose) recommended by the EFSA.

**Table 3:** Tolerable durations of exposure to non-carcinogenic LSTs according to the consumption and contamination data.

Toxin Group	Scenario	Mean Contamination Data	Consumption Data		Exposure Duration (Years)
		Values (µg/kg)	Type	Values (g/d)	
OA	Scenario 1 ^1^	0.2 (LB ^3^)–26.0 (UB ^4^)	mean	2.8	19.7–2839.3
	Scenario 2 ^2^		P95	11.4	4.9–700.4
PTX2	Scenario 1	0.05 (LB)–10.0 (UB)	mean	2.8	136.7–28,742.4
	Scenario 2		P95	11.4	33.7–7090.5
YTX	Scenario 1	13.4 (LB)–50.4 (UB)	mean	2.8	848.3–3186.2
	Scenario 2		P95	11.4	209.3–786

^1^ Scenario 1, mean shellfish consumption, mean contamination level: LB–UB. ^2^ Scenario 2, the 95th percentile shellfish consumption, mean contamination level: LB–UB. ^3^ LB, ND = 0; ^4^ UB, ND = LOD.

**Table 4:** Hazard index (HI) for dietary exposure to LSTs alone and LSTs plus PSTs calculated as the sum of the deterministically estimated %ARfD values.

Age (Years)	HI (%) for LSTs ^1^ LB ^2^–UB ^3^		HI (%) for LSTs Plus PSTs ^4^ LB–UB	
	Scenario 1 ^5^	Scenario 2 ^6^	Scenario 1	Scenario 2
All	12.7–20.7	0.5–15.2	68.7–**106.7**	2.5–55.2
≤6	28.7–46.6	1.1–34.3	152.7–**240.6**	3.1–**124.3**
7–13	14.3–23.2	0.5–17.1	76.3–**119.2**	2.5–61.1
14–17	11.7–19.1	0.4–14.0	63.7–99.1	0.4–50.0
18–59	12.8–20.8	0.5–15.4	68.8–**106.8**	2.5–55.4
≥60	9.9–16.1	0.4–11.8	51.9–82.1	0.4–41.8

^1^ HI (%) for LSTs, the sum of the %ARfD values of toxins in the OA, PTX, and YTX groups, with values > 100% in bold; ^2^ LB, ND = 0; ^3^ UB, ND = LOD. ^4^ HI (%) for LSTs plus PSTs, the sum of the %ARfD values of LSTs and PSTs, with data on the exposure to PSTs from our previously published article [59], with values > 100% in bold. ^5^ Scenario 1 (acute exposure): the 95th percentile (P95) daily shellfish consumption and maximum concentration; ^6^ Scenario 2: the 95th percentile (P95) daily shellfish consumption and concentration (3.5 (LB)–134.0 (UB) µg STX eq./kg for PSTs; 0 (LB)–26.0 (UB) µg OA eq./kg, 0 (LB)–10 (UB) µg PTX2 eq./kg, and 79.5 (LB)–99.5 (UB) µg YTX eq./kg for LSTs).

**Table 5:** Toxicity equivalence factors (TEFs) and regulatory limits for LSTs.

Toxin Group	Toxin	TEF ^1^	Regulatory Limit ^2^
OA	OA *	1	160 µg OA eq./kg
	DTX1	1	
	DTX2	0.6	
PTX	PTX2 *	1	deregulated
YTX	YTX *	1	3.75 mg YTX eq./kg
	HomoYTX	1	

*—Index compounds; ^1^ The TEFs for each LST group were as per the EFSA regulations [29,30,33]; ^2^ EU limits for each LST group [47,48].

## Data Availability

The data presented in this study are available upon request from the corresponding author due to restrictions, e.g., privacy or ethical.

## Supplementary Materials

The following supporting information can be downloaded at: https://www.mdpi.com/article/10.3390/md22060239/s1, Table S1: Detection rates and concentrations of OA group toxins in samples; Table S2: Detection rates and concentrations of PTX group toxins in samples; Table S3: Detection rates and concentrations of YTX group toxins in samples.
