# Supplementary material for: Occurrence and Exposure Assessment of Lipophilic Shellfish Toxins in the Zhejiang Province, China
Source: Mar Drugs. 2024 May 24;22(6):239. doi: 10.3390/md22060239 (PMC11205204; doi:10.3390/md22060239)
Supplement: Supplementary file 1 [file marinedrugs-22-00239-s001.zip › supplementary table.pdf]

Table S1. Detection rates and concentrations of OA group toxins in samples.

		Sample size	N[1]	Percentage (%[2])	Mean[3] (μg OA eq./kg)		Median[4] (μg OA eq./kg)		Max[5] (μg OA eq./kg)	
					LB[6]	UB[7]	LB	UB	LB	UB
	Total species	546	8	1.5	0.2	26.0	0.0	26.0	16.5	32.5
Species										
	Atrina pectinata	2	0	0.0	0.0	26.0	0.0	26.0	0.0	26.0
	Scapharca subcrenata	24	0	0.0	0.0	26.0	0.0	26.0	0.0	26.0
	Arcidae (Except for *Scapharca subcrenata*)	53	0	0.0	0.0	26.0	0.0	26.0	0.0	26.0
	Oysters	77	0	0.0	0.0	26.0	0.0	26.0	0.0	26.0
	Scallops	36	0	0.0	0.0	26.0	0.0	26.0	0.0	26.0
	Mussels	354	8	2.3	0.3	26.1	0.0	26.0	16.5	32.5
Sampling time										
2018	May	30	0	0.0	0.0	26.0	0.0	26.0	0.0	26.0
2018	June	123	0	0.0	0.0	26.0	0.0	26.0	0.0	26.0
2018	July	40	0	0.0	0.0	26.0	0.0	26.0	0.0	26.0
2018	August	40	1	2.5	0.4	26.2	0.0	26.0	16.5	32.5
2018	September	48	0	0.0	0.0	26.0	0.0	26.0	0.0	26.0
2019	May	70	0	0.0	0.0	26.0	0.0	26.0	0.0	26.0
2019	June	134	7	5.2	0.6	26.1	0.0	26.0	16.0	32.0
2019	July	28	0	0.0	0.0	26.0	0.0	26.0	0.0	26.0
2019	August	10	0	0.0	0.0	26.0	0.0	26.0	0.0	26.0
2019	September	23	0	0.0	0.0	26.0	0.0	26.0	0.0	26.0
Sampling site										
	Hangzhou	29	5	17.2	2.1	26.4	0.0	26.0	16.0	32.0
	Ningbo	124	3	2.4	0.3	26.1	0.0	26.0	16.5	32.5
	Taizhou	124	0	0.0	0.0	26.0	0.0	26.0	0.0	26.0
	Wenzhou	145	0	0.0	0.0	26.0	0.0	26.0	0.0	26.0
	Zhoushan	124	0	0.0	0.0	26.0	0.0	26.0	0.0	26.0

[1] N, the number of samples contaminated with LSTs;
[2] %, the percentage of samples contaminated with LSTs;
[3] Mean, arithmetic mean;
[4] Median, the 50th percentile;
[5] Max, the maximum value in a group;
[6] LB, ND = 0;
[7] UB, ND = LOD.

Table S2. Detection rates and concentrations of PTX group toxins in samples.

		Sample size	N[1]	Percentage (%[2])	Mean[3] (µg PTX eq./kg)		Median[4] (µg PTX eq./kg)		Max[5] (µg PTX eq./kg)	
					LB[6]	UB[7]	LB	UB	LB	UB
	Total species	546	2	0.4	0.05	10.0	0.0	10.0	13.0	13.0
Species										
	Atrina pectinata	2	0	0.0	0.0	10.0	0.0	10.0	0.0	10.0
	Scapharca subcrenata	24	0	0.0	0.0	10.0	0.0	10.0	0.0	10.0
	Arcidae (Except for *Scapharca subcrenata*)	53	0	0.0	0.0	10.0	0.0	10.0	0.0	10.0
	Oysters	77	1	1.3	0.2	10.0	0.0	10.0	13.0	13.0
	Scallops	36	0	0.0	0.0	10.0	0.0	10.0	0.0	10.0
	Mussels	354	1	0.3	0.04	10.0	0.0	10.0	13.0	13.0
Sampling time										
2018	May	30	0	0.0	0.0	10.0	0.0	10.0	0.0	10.0
2018	June	123	2	1.6	0.2	10.0	0.0	10.0	13.0	13.0
2018	July	40	0	0.0	0.0	10.0	0.0	10.0	0.0	10.0
2018	August	40	0	0.0	0.0	10.0	0.0	10.0	0.0	10.0
2018	September	48	0	0.0	0.0	10.0	0.0	10.0	0.0	10.0
2019	May	70	0	0.0	0.0	10.0	0.0	10.0	0.0	10.0
2019	June	134	0	0.0	0.0	10.0	0.0	10.0	0.0	10.0
2019	July	28	0	0.0	0.0	10.0	0.0	10.0	0.0	10.0
2019	August	10	0	0.0	0.0	10.0	0.0	10.0	0.0	10.0
2019	September	23	0	0.0	0.0	10.0	0.0	10.0	0.0	10.0
Sampling site										
	Hangzhou	29	0	0.0	0.0	10.0	0.0	10.0	0.0	10.0
	Ningbo	124	0	0.0	0.0	10.0	0.0	10.0	0.0	10.0
	Taizhou	124	0	0.0	0.0	10.0	0.0	10.0	0.0	10.0
	Wenzhou	145	2	1.4	0.2	10.0	0.0	10.0	13.0	13.0
	Zhoushan	124	0	0.0	0.0	10.0	0.0	10.0	0.0	10.0

[1] N, the number of samples contaminated with LSTs;
[2] %, the percentage of samples contaminated with LSTs;
[3] Mean, arithmetic mean;
[4] Median, the 50th percentile;
[5] Max, the maximum value in a group;
[6] LB, lower bound, ND = 0;
[7] UB, upper bound, ND = LOD.

Table S3. Detection rates and concentrations of YTX group toxins in samples.

		Sample size	N[1]	Percentage (%[2])	Mean[3] (μg YTX eq./kg)		Median[4] (μg YTX eq./kg)		Max[5] (μg YTX eq./kg)	
					LB[6]	UB[7]	LB	UB	LB	UB
	Total species	546	82	15.0	13.4	50.4	0.0	40.0	373.0	393.0
Species										
	Atrina pectinata	2	0	0.0	0.0	40.0	0.0	40.0	0.0	40.0
	Scapharca subcrenata	24	0	0.0	0.0	40.0	0.0	40.0	0.0	40.0
	Arcidae (Except for *Scapharca subcrenata*)	53	0	0.0	0.0	40.0	0.0	40.0	0.0	40.0
	Oysters	77	0	0.0	0.0	40.0	0.0	40.0	0.0	40.0
	Scallops	36	3	8.3	5.0	43.3	0.0	40.0	80.6	100.6
	Mussels	354	79	22.3	20.2	55.7	0.0	40.0	373.0	393.0
Sampling time										
2018	May	30	10	33.3	30.9	64.3	0.0	40.0	275.0	295.0
2018	June	123	22	17.9	22.6	59.0	0.0	40.0	334.0	354.0
2018	July	40	2	5.0	1.5	40.5	0.0	40.0	32.0	52.0
2018	August	40	2	5.0	3.3	42.3	0.0	40.0	72.8	92.8
2018	September	48	22	45.8	29.2	60.0	0.0	40.0	132.0	152.0
2019	May	70	1	1.4	0.3	40.0	0.0	40.0	20.3	40.3
2019	June	134	23	17.2	15.0	51.6	0.0	40.0	373.0	393.0
2019	July	28	0	0.0	0.0	40.0	0.0	40.0	0.0	40.0
2019	August	10	0	0.0	0.0	40.0	0.0	40.0	0.0	40.0
2019	September	23	0	0.0	0.0	40.0	0.0	40.0	0.0	40.0
Sampling site										
	Hangzhou	29	14	48.3	21.9	52.2	0.0	40.0	68.9	88.9
	Ningbo	124	15	12.1	5.9	43.5	0.0	40.0	113.0	133.0
	Taizhou	124	14	11.3	8.3	46.0	0.0	40.0	132.0	152.0
	Wenzhou	145	15	10.3	15.6	53.5	0.0	40.0	373.0	393.0
	Zhoushan	124	24	19.4	21.6	57.8	0.0	40.0	334.0	354.0

[1] N, the number of samples contaminated with LSTs;
[2] %, the percentage of samples contaminated with LSTs;
[3] Mean, arithmetic mean;
[4] Median, the 50th percentile;
[5] Max, the maximum value in a group;
[6] LB, ND = 0;
[7] UB, ND = LOD.
